# Case Report: Bilateral tonic-clonic seizures as initial presentation of Lhermitte–Duclos Disease and Cowden Syndrome

**DOI:** 10.3389/fonc.2026.1950323

**Published:** 2026-09-25

**Authors:** Winston Tse-Hou Kwok, Inci Yaman Bajin, Seth Andrew Climans

**Affiliations:** 1 Schulich School of Medicine and Dentistry, Western University, London, ON, Canada; 2 Department of Oncology, Western University, London, ON, Canada; 3 London Health Sciences Centre, London, ON, Canada

**Keywords:** Cowden syndrome, epilepsy, high-field MRI, Lhermitte-Duclos disease, PTEN

## Abstract

Lhermitte–Duclos Disease is a rare, benign cerebellar tumour that is a manifestation of Cowden Syndrome, a hereditary tumour predisposition syndrome caused by germline mutations in the *PTEN* gene. Here, we report a 38-year-old female patient with no past medical history or family neurological history presenting to hospital with a new, unprovoked, bilateral tonic-clonic seizure with no apparent seizure focus. EEG showed no epileptiform activity, and 7T MRI demonstrated bilateral Lhermitte–Duclos Disease as well as several cortical meningoencephaloceles. Her seizures were controlled with levetiracetam, and given her cerebellar lesion was minimally symptomatic, no further interventions were pursued. *PTEN* germline testing identified a heterozygous c.1003C>T (p.Arg335*) variant known to be pathogenic for Cowden Syndrome, and we promptly referred this patient for cancer screening. She was diagnosed with ductal carcinoma *in situ* and thyroid atypia of undetermined significance, and she subsequently underwent bilateral mastectomy and total thyroidectomy respectively. Lhermitte–Duclos Disease and Cowden Syndrome may present initially in previously healthy patients with bilateral tonic-clonic seizures without clear epileptogenic foci. Recognition of the underlying hereditary cancer syndrome enables timely cancer detection, potentially improving treatment outcomes.

## Introduction

Lhermitte–Duclos Disease (LDD), or dysplastic cerebellar gangliocytoma, is a rare, benign cerebellar tumour that affects all age groups, though it typically presents between 30 to 40 years of age (1). Patients may be asymptomatic or present clinically with cerebellar signs such as ataxia, dysarthria, dysmetria, and dysdiadochokinesia as well as symptoms of raised intracranial pressure, including headaches, nausea, or visual disturbances (2–4). MRI is the imaging modality of choice for LDD, which would feature enlarged and disorganized cerebellar folia with hyperintense signal striations in a characteristic “tiger-stripe” appearance (5).

LDD is associated with germline mutations in the tumour suppressor gene *PTEN*, and it is considered one of the manifestations of Cowden Syndrome (CS), an autosomal dominant, hereditary cancer predisposition syndrome caused by germline *PTEN* mutations (6, 7). CS has an estimated prevalence of 1 in 200,000. Patients with CS may develop characteristic mucocutaneous hamartomatous lesions and have increased risk of developing breast, thyroid, kidney, colorectal, and endometrial cancers, hence requiring an enhanced cancer surveillance strategy compared to the general population (6, 8).

Here, we report a case in which bilateral tonic-clonic seizures were the initial presentation of LDD and CS in an otherwise healthy patient, with the diagnoses established with 7T MRI and *PTEN* molecular testing on subsequent evaluation. Timely recognition of the underlying hereditary cancer syndrome following patient’s initial neurological presentation enabled the early detection and treatment of the patient’s breast and thyroid cancers.

## Case report

### Case description

A 38-year-old, previously healthy woman presented to our centre after experiencing a first bilateral tonic-clonic seizure of unknown whether focal or generalized onset. She had no recollection of the event, and per collateral history, there were no focal neurological deficits in the post-ictal phase. There was no history of prodrome, headaches, CNS infections, head trauma, previous seizures, metabolic disturbances, or toxic ingestion. She was not particularly coordinated or athletic, but she had unremarkable neurological development. There was no family history of epilepsy or neurodevelopmental disorders. She worked as an educator and lived at home. She was afebrile, and physical examination revealed only mild macrocephaly, mild ataxic dysarthria, and subtle difficulties performing tandem gait. There were no focal neurological deficits, dysmetria, or dysdiadochokinesia. Laboratory investigations did not show any electrolyte abnormalities. On CT Head, there were no structural lesions within the cortex, but there were low-lying cerebellar tonsils, mild compression of the 4th ventricle, and ventriculomegaly with sulcal effacement, suggesting slightly raised intracranial pressure.

There were no obvious causes to the patient’s seizures, including structural abnormalities, infectious or autoimmune encephalitis, toxic ingestion, or metabolic derangement. However, given the imaging findings, it was felt that our patient was at higher risk of recurrent seizures. Hence, she was initiated on levetiracetam 500 mg twice daily and subsequently discharged with arrangements for EEG, MRI, and outpatient follow-up.

Patient’s EEG showed a non-specific finding of mild diffuse slowing with no epileptiform activity. She underwent a 1.5T MR Head followed by a 7T MR Head. Compared to the 1.5T MRI, 7T MRI better demonstrated an enlarged cerebellum with bilaterally distorted folia, two mass-like lesions localized to the left and right cerebellar hemispheres, and striated bands of hyperintense signal in a tiger-stripe pattern seen on T2-FLAIR (**Figure 1**). On susceptibility-weighted images, there was a punctate focus of mineralization in the superior cerebellar vermis. These findings were concerning for bilateral Lhermitte-Duclos Disease (LDD), although pediatric-type low-grade glioma and, less likely, cerebellitis and cerebellar infarct remained within the differential diagnosis. Mass effect from this lesion led to stable, compensated hydrocephalus, with the cerebellar tonsils descending 1.5 cm below the foramen magnum. Within the cortex, there was no definite cortical dysplasia. There was a small meningocele at the anterior pole of the right temporal lobe (**Figure 2**) and as well as small bilateral temporal encephaloceles extending into the greater sphenoid wings.

**Figure 1:**
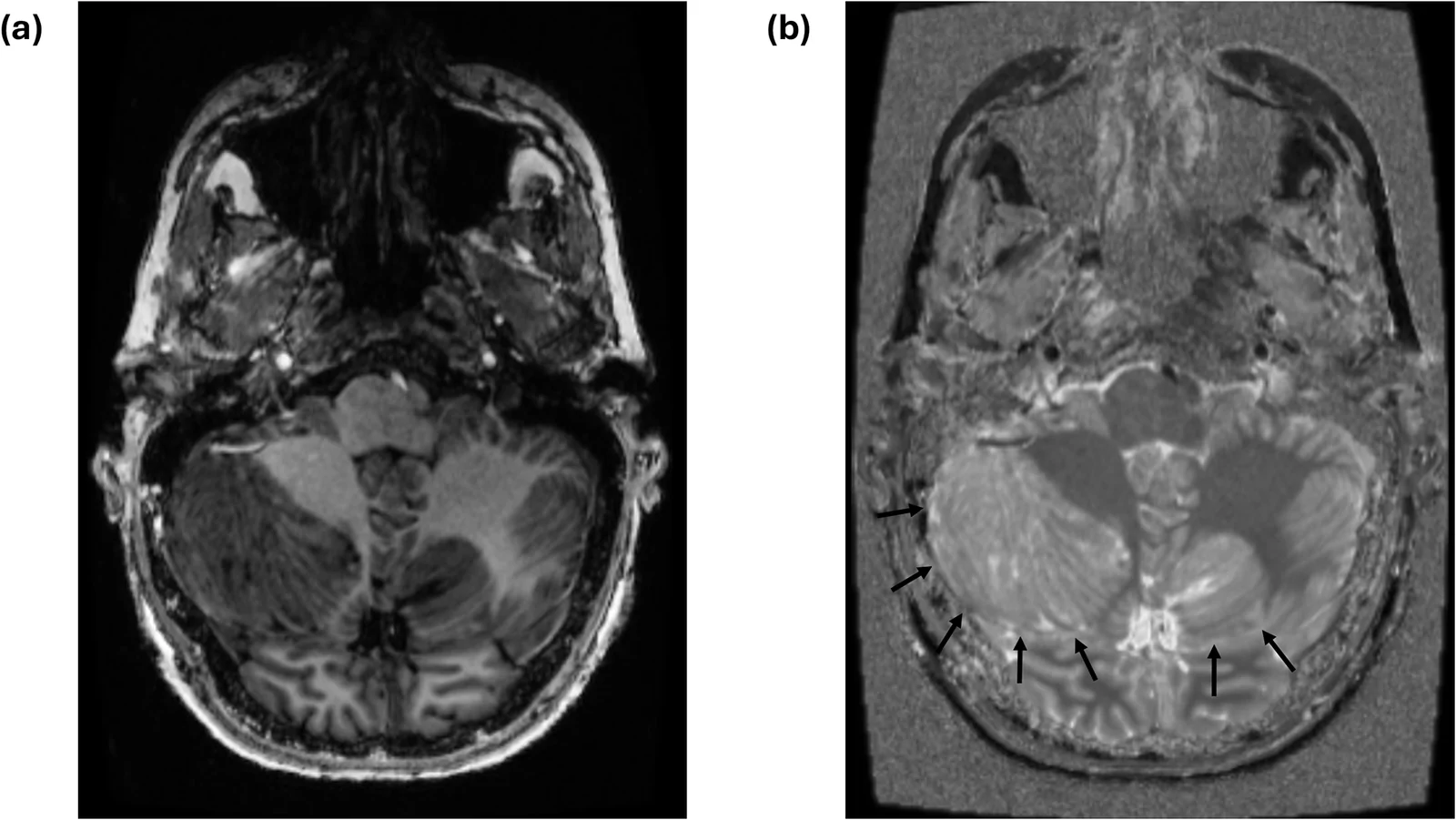
7T MR head image demonstrating bilateral Lhermitte-Duclos disease. **(a)** T1-weighted axial 7T MRI without gadolinium at the level of the cerebellum. **(b)** T2-weighted axial 7T MRI at the same level. Arrows point toward bilateral cerebellar lesions, larger on the right than left, with a tiger-stripe pattern suggestive of Lhermitte-Duclos Disease.

**Figure 2:**
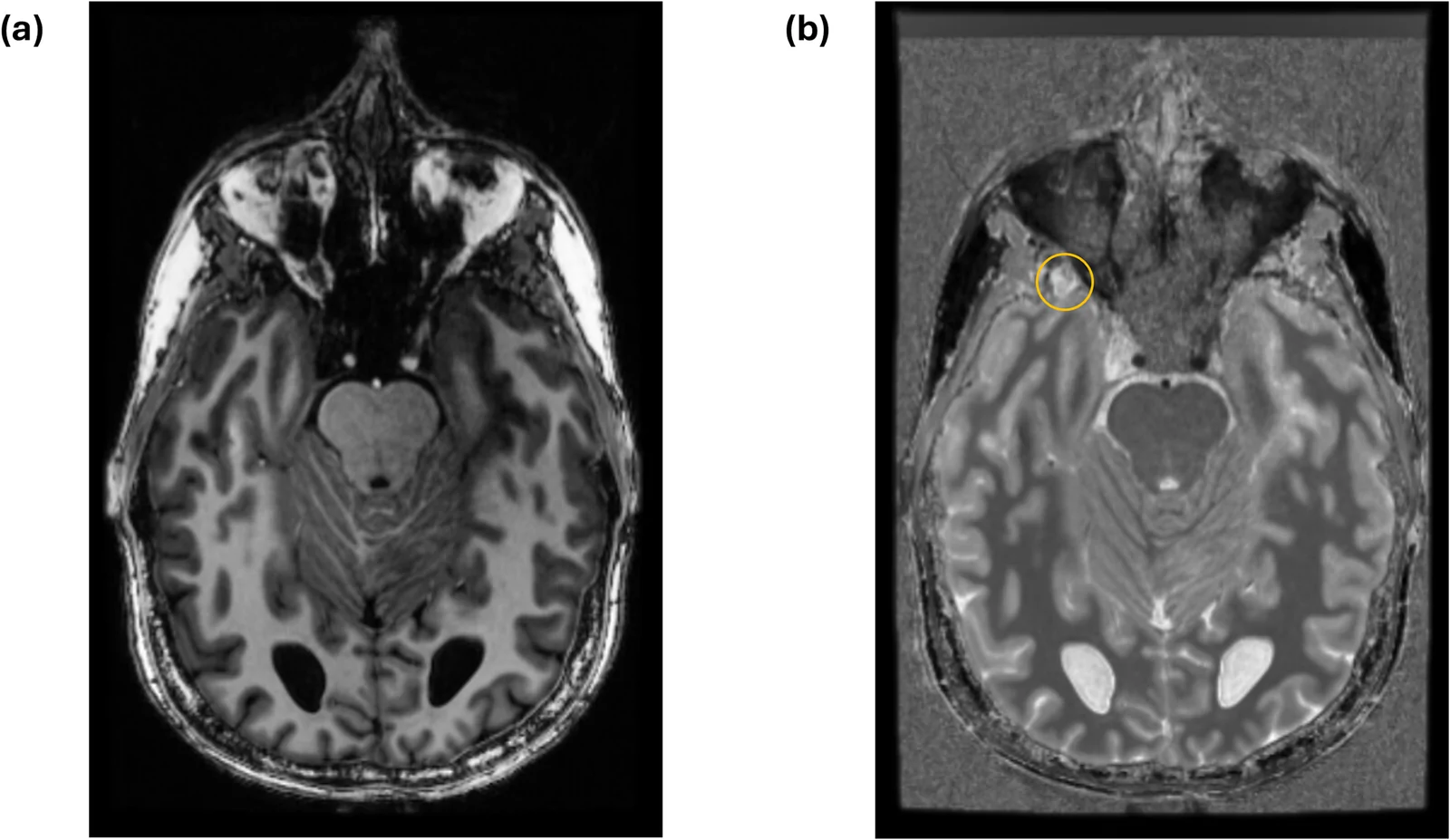
7T MR head image demonstrating a right temporal pole meningocele. **(a)** T1-weighted axial 7T MRI without gadolinium and **(b)** T2-weighted axial 7T MRI at the same level, demonstrating a meningocele at the anterior pole of right temporal lobe, indicated by the annotated circle. The bilateral polar encephaloceles are not visualized at this level.

Following initial presentation to hospital for her seizures, our patient remained on levetiracetam for 6 months. She underwent a trial of levetiracetam discontinuation given the non-specific findings on EEG. Unfortunately, her seizures recurred 6 months following levetiracetam discontinuation, and levetiracetam was reinitiated. This was well-tolerated with no side effects. Additionally, as our patient was minimally asymptomatic from her cerebellar lesion and hydrocephalus, neurosurgical intervention was deemed not necessary at this time, and annual MRI surveillance of the cerebellar lesions was opted for instead.

Given high suspicion for LDD and CS, we proceeded with germline testing for mutations in *PTEN*. High-throughput sequencing identified a heterozygous variant in exon 8, c.1003C>T (p.Arg335*), previously reported to be pathogenic for CS, confirming the diagnosis of LDD and CS. Further interview revealed that the patient’s mother, currently in her 60s, has advanced breast cancer, her maternal grandfather was diagnosed with colon cancer in his 50s, and her brother underwent a total thyroidectomy for unclear thyroid disease, altogether suspicious for a familial tumour predisposition syndrome. With this, we promptly referred our patient to specialists in Medical Genetics, Surgical Oncology, Endocrinology, and Otolaryngology for further assessment and management. Subsequent breast cancer screening with follow-up biopsy identified right ductal carcinoma *in situ*, and thyroid ultrasound identified a TIRADS 4 nodule in the right thyroid lobe with fine needle aspiration demonstrating atypia of undetermined significance. At the time of writing, our patient underwent a bilateral mastectomy and total thyroidectomy, both of which were uncomplicated and well-tolerated. Final pathology results for her thyroid tissue remain pending, but there has been no evidence of nodal or metastatic disease.

### Follow-up and outcomes

The total duration of follow-up from initial presentation to manuscript writing is 33 months. While our patient had her driving licence was suspended following her second seizure, she has remained seizure free for 23 months with re-initiation of levetiracetam therapy, and she has been able to return to her usual activities since. She continues to receive annual MR Head for surveillance of her LDD, which has remained stable within the follow-up period. Additionally, she is undergoing active surveillance for renal and colorectal cancers, and she is awaiting her dermatological assessment for melanoma screening. Moreover, her brother also tested positive for a germline *PTEN* mutation, and genetic counselling was provided. While the diagnosis of Cowden Syndrome in our patient has been challenging and impacting her quality of life, particularly given the uncertainty associated with her increased risk of cancer, she has been actively engaging in counselling and wellness, which has been helpful for her psychological well-being.

### Clinical timeline

Timeline of patient’s clinical course is outlined in **Figure 3**.

**Figure 3:**
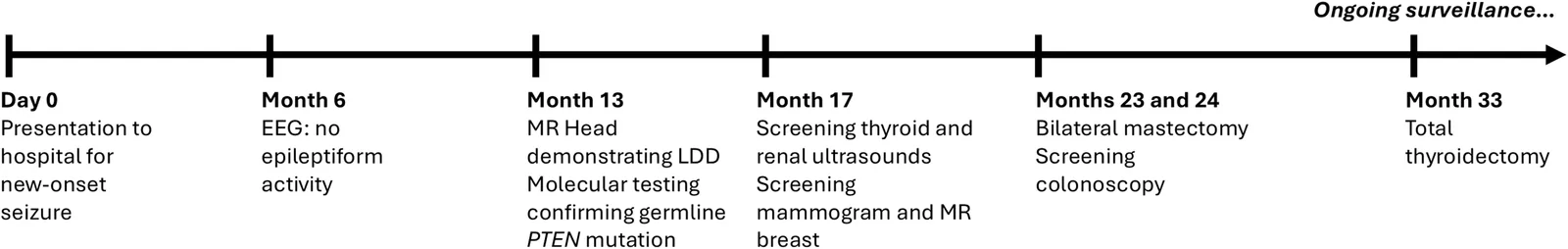
Timeline of patient’s clinical course from initial presentation to time of manuscript writing.

## Discussion

Lhermitte-Duclos Disease (LDD), or dysplastic cerebellar gangliocytoma, is a rare, slow-growing cerebellar tumour classified as a WHO Grade 1 glioneuronal and neuronal tumour, though some authors have suggested that LDD also demonstrates hamartomatous features (1, 9, 10). Histologically, it is characterized by infiltration and replacement of the granule cell layer by hypertrophic ganglion cells, loss of Purkinje neurons, and hypermyelination of the molecular layer, which is proposed to be a result of aberrant cell signalling due to *PTEN* loss of function (11–13). LDD may be managed expectantly in asymptomatic patients. Surgical resection of the lesion or CSF diversion may be pursued in patients with symptoms of cerebellar dysfunction or raised intracranial pressure, and there have been rare cases of tumour recurrence following partial tumour resection (3, 14–17). There is limited evidence for chemotherapy or radiation therapy in the treatment of LDD. A strong association between LDD and Cowden Syndrome has been well described, with Padberg et al. first proposing that the two represent a single clinical entity in 1991 (18, 19).

Cowden Syndrome (CS), along with Bannayan-Riley-Ruvalcaba syndrome (BRRS) and Proteus-like syndrome, belongs to a heterogeneous group of disorders involving germline *PTEN* mutations known as *PTEN* hamartoma tumor syndromes (PHTS) (20). *PTEN*, a tumour suppressor gene located on chromosome 10, encodes an inhibitory phosphatase in the PI3K-Akt-mTOR signalling pathway, thereby regulating cell proliferation, survival, and migration (21). Manifestations of CS are multisystem and highly diverse, arising from significant pleiotropy associated with germline PTEN mutations (22–24). Skin lesions including trichilemmomas, acral keratoses, and papillomatous papules alongside gastrointestinal polyps with potential for malignant transformation are prevalent in patients with CS (25, 26). Additionally, CS is associated with significantly elevated lifetime risks of developing breast, thyroid, endometrial, kidney, and colorectal cancers (27). Recent estimates report up to 91% lifetime risk of developing breast cancer and median age at breast cancer diagnosis of 40 years (28, 29). Among neurologic manifestations of CS, LDD is considered pathognomonic, whereas other presentations commonly reported include macrocephaly, developmental delay, and autism spectrum disorder (6, 30–32). Rare cases of epilepsy have also been reported in patients with CS, though these have been in context of cortical dysplasia or neurodevelopmental disorders (33–36). **Table 1** outlines the current proposed diagnostic criteria for CS.

**Table 1:** Diagnostic criteria for Cowden syndrome/PTEN hamartoma tumour syndrome, revised 2013.

Major criteria	Minor criteria
Breast cancer Endometrial cancer (epithelial) Thyroid cancer (follicular) ≥3 gastrointestinal hamartomas or ganglioneuromas Lhermitte-Duclos disease (adult) Macrocephaly, ≥97 percentile (58 cm in adult female, 60 cm in adult male) Macular pigmentation of glans penis Mucocutaneous lesions • ≥3 Trichilemmoma (at least 1 biopsy-proven) • ≥3 palmoplantar keratotic pits and/or acral hyperkeratotic papules) • ≥3 mucocutaneous neuromas • ≥3 or biopsy proven oral papilloma (particularly on tongue and gingiva)	Autism spectrum disorder Colon cancer ≥3 esophageal glycogenic acanthoses ≥3 lipomas Intellectual disability (i.e. IQ ≤75) Renal cell carcinoma Testicular lipomatosis Papillary or follicular variant of papillary thyroid cancer Thyroid structural lesions (e.g. adenoma, nodule, goiter) Vascular anomalies (including multiple intracranial developmental venous anomalies)
Must have: • 3 or more major criteria, and 1 must include Macrocephaly, Lhermitte-Duclos disease, or gastrointestinal hamartomas; or • 2 major and 3 minor criteria.	

Adapted with permission from Pilarski et al, 2013 (6).

Given the wide-reaching implications of CS, we advocate for a collaborative, interdisciplinary approach from diagnosis to management to optimize patient outcomes. CS may be suspected in patients presenting with a first bilateral tonic-clonic seizure and subtle cerebellar signs in the absence of neurodevelopmental or oncological history or clear epileptogenic foci, as illustrated in our case. High-field MRI and *PTEN* molecular testing can facilitate diagnosis of LDD and CS, enabling timely referral to appropriate specialists for cancer surveillance and intervention. The National Comprehensive Cancer Network (NCCN) recommends a multi-system cancer surveillance protocol for patients with CS (37). This includes annual mammography and breast MRI for breast cancer screening beginning at age 30 years (or 10 years before the earliest known breast cancer within the family), annual thyroid ultrasound starting at age 12, annual dermatologic examination for melanoma, screening colonoscopy at least every 5 years starting at age 35 (or 5–10 years before earliest known colorectal cancer within the family), annual renal ultrasound beginning at age 40, and patient education regarding abnormal uterine bleeding for endometrial cancer surveillance along with discussion of possible risk-reducing hysterectomy.

The exact origin of our patient’s seizures, albeit they were adequately managed with levetiracetam, remains uncertain. Cerebellar lesions such as LDD typically do not cause seizures, and there were no lateralizing or localizing findings on our patient’s EEG. We suspect her seizures may be due to her small left anterior temporal meningocele or small temporal encephaloceles visualized only on her 7T MRI and not on 1.5T MRI, or due to tiny cortical hamartomas or other substrates that cannot be visualized with current imaging techniques, as recently proposed by Serôdio et al (38). Hence, the use of 7T MRI in this study supports the growing role of ultra-high field neuroimaging in clinical practice. Additionally, to our knowledge, this is the second published 7T MR image of LDD (39).

It is important to recognize a few limitations of this case. First, the diagnosis of LDD in this case was based on MRI and germline *PTEN* mutation testing without histopathological confirmation of the lesion. Second, the follow-up period of 33 months is relatively limited given the benign, slow-growing nature of LDD. Third, this case is inherently limited in its generalizability as it is a single case report. However, this case is valuable as it highlights seizures as a potential presentation of Cowden Syndrome, which may prompt a multidisciplinary approach in further evaluation and management.

## Patient perspective

Growing to the age of 39, I hadn’t ever had any medical challenges. I was “walking” through life normally, I was “healthy”.

When I attended Community College, I learned about seizures how to support someone through one, what to look for etc. and when to seek further medical assistance. Before my first seizure, I felt no different than I had any other day. Hearing about it afterward and the difficulties that I was now experiencing were worrisome, I knew that something really wasn’t right. My world from where I knew things to be was completely upended.

The diagnosis is/was a heavy one. In time, I try to learn what I can at a time that is appropriate for me, when my brain can handle it, so as to not get stuck in the doom and gloom of this medically complex condition. Understanding that everyone is medically unique and experiences things differently, has also taken a long time to process, there is no “one stop” to treating this. I try and seek answers from trusted sources as MUCH as I can taking notes at appointments and having someone attend with me so I’m able to draw from that knowledge later. Ending up down a Google rabbit hole isn’t helpful to anyone and perhaps what I’m reading doesn’t even pertain to me. I have sought support from Mental Health professionals when the gravity of this situation is simply “just too much.” In the meantime, life has continued. I try and eat well while remaining active, making a point to do things that make me happy.

## Data Availability

The original contributions presented in the study are included in the article/Supplementary Material, further inquiries can be directed to the corresponding author.
